# The Book World of Medicine and Science

**Published:** 1898-06-25

**Authors:** 


### THE BOOK WORLD OF MEDICINE AND SCIENCE.

A System of Medicine by Many Writers. Edited by Thomas Clifford Allbutt, M.D., F.R.S., F.R.C.P. Regius Professor of Physic in the University of Cambridge. Volume V. (London: Macmillan and Co. 1898. Pp. 1,058; illustrated. Price 25s. net.)

First Notice.

This handsome volume is very welcome, dealing as it does with a group of diseases which form the backbone of the daily work of the English practitioner. Diseases of the respiratory organs, of the pleura, and of the circulatory system are the subjects included. Bronchitis is treated of by Dr. Ewart. In classifying cases of this disease a useful distinction is drawn between bronchitis of the smaller tubes and capillary bronchitis, the former being the condition met with in the acute suffocative bronchitis of adults, while the latter—an inflammation of the sublobular and lobular bronchi extending into the alveoli—is the form found in infancy and old age. This is an anatomical difference which no doubt explains the fact that while in the one form expectoration tends to be suppressed, in the other it continues. The article on "Bronchiectasis," also by Dr. Ewart, is a very interesting one, partly from the care with which the physical problems connected with the accumulation of fluids in cavities connected with bronchial tubes are discussed, and partly from the description given of that generalised condition of bronchiectasis of the smaller tubes—bronchiolectasis as it is called—which is apt to occur as an acute disease in children, especially in connection with the bronchitis of measles and whooping-cough. Dr. Pye Smith treats fully of pneumonia, appealing largely, as is only natural, to personal and to Guy's experience. In dealing with catarrhal pneumonia, he, to a certain extent, goes over the same ground as the preceding articles. The description of cirrhosis of the lung is short, but is probably sufficient. Phthisis pulmonalis is what one may call a big subject, and is well treated by Dr. Percy Kidd in an article of nearly ninety pages. While full recognition is given to the infective nature of tuberculosis, Dr. Kidd does not fail to show the influence exercised by the so-called predisposing causes. The article is very carefully written, and perhaps almost too free from dogmatism; but the reader will probably rise from a perusal of it impressed with the opinion that "house infection" is at the bottom of much of the phthisis which we so commonly meet with. In regard to treatment, Dr. Percy Kidd—moved probably thereto by a large and long experience among out-patients and by the knowledge thus borne in upon him that the great mass of the sufferers from the disease are quite unable to obtain the advantages of climatic and "cure" treatment—has given a large amount of space to a careful consideration of the medicinal means which are at our disposal in dealing with the disease. This part of the article will be read with much pleasure by practical men, for it is to be feared that it has become far too much the custom of late to look upon every treatment, except that by climate, hygiene, and fresh air, as a mere placebo. As an introduction to diseases of the pleura, Dr. Samuel West gives a chapter on intra-pleural tension, which, taken as a whole, is not very convincing. The article by Dr. Gee and Dr. Herringham is distinctly good. Very properly, emphasis is laid on the liability to unexpected and speedy death which hangs over cases of pleural effusion, even when it is only serous. As to treatment, it is succinctly stated that "the treatment of pleurisy with effusion relates almost wholly to removal of the effusion," and although other measures are described, the modern attitude is well expressed by saying: "But the question of paracentesis is always foremost in the mind," incision and drainage in cases of empyema, of course, included in the term. Perhaps, however, the author is hardly justified in drawing such a definite line, as he does, between serous and purulent effusion in regard to treatment, and in saying that in serous cases, even when the effusion continues, "drainage by a permanent opening is out of the question." The persistent recurrence of effusion, even of clear fluid, is often so grave a matter that mere puncture, repeated again and again, can hardly be regarded as the last word that can be said as to treatment.

Radiography. By S. R. Bottone. (London and New York. Whittaker and Co. 1898. Pp. 172. Illustrations and diagrams. Price 3s.)

This is one of the many books now appearing which relate to that discovery of Professor Röntgen's which, more than any scientific achievement of late years, has strongly attracted the popular mind. Seldom has application and exploitation followed so quickly on the heels of scientific discovery as in the case of the Röntgen rays. Yet the discovery itself was the result of years of patient work, conducted, by many investigators, with the single eye of science and with little thought of practical utilisation. Mr. Bottone's book does not deal with the uses of radiography so much as with the technical details, constructional and manipulative, a knowledge of which is necessary if the best results are to be obtained. There is given a sufficiency of theory to render the use of the necessary apparatus an intelligent proceeding; and abundant and precise directions for constructing the apparatus itself. Practical as the author is, he is not one of those practical men who glory in "doing things without knowing the reason why." Mr. Bottone is a firm believer in the superiority, for X-ray purposes, of the Wimshurst machine over any coil, though he cordially recognises the excellence of the work done by Dr. Monell with the Holtz machine. For use with a Wimshurst Mr. Bottone prefers a Jackson focus tube, and in the matter of fluorescence he pins his faith to barium ferrocyanide. In the last chapter the author briefly discusses the chief views held as to the nature of the Röntgen rays, and, differing from others, unfolds a "working hypothesis" of his own. But, whatever the value of this hypothesis may prove to be, the author is to be congratulated on having produced an excellent and reliable guide to a very fascinating pursuit.

Medicated Wines and Their Dangers. By Walter N. Edwards, F.C.S. (London: 1898. The Women's Total Abstinence Union. Pp. 29. Price 2d., or 12s. per 100.)

This pamphlet is introduced by a likeness of the author in the attitude of delivering a science lecture, with the aid of some very complicated apparatus arranged upon a highly-elaborate tablecloth. It concludes with an advertisement of the unfermented wines supplied by a certain firm; sandwiched between such an exordium and peroration is a mass of details as to the percentage of alcohol in various medicinal preparations and much expression of perturbation as to the effects of their extended use as beverages. With the principle of the brochure, the exposure of the evils resulting from addiction to alcohol, chloral, morphia, cocaine, &c., we are entirely in accord, but we venture to doubt whether the good cause will be really advanced by the method of presentation therein exemplified.

